# Supplementary material for: Superspreaders drive the largest outbreaks of hospital onset COVID-19 infections
Source: eLife. 2021 Aug 24;10:e67308. doi: 10.7554/eLife.67308 (PMC8384420; doi:10.7554/eLife.67308)
Supplement: Transparent reporting form [file elife-67308-transrepform.docx]

***eLife’s* transparent reporting form**

We encourage authors to provide detailed information *within their submission* to facilitate the interpretation and replication of experiments. Authors can upload supporting documentation to indicate the use of appropriate reporting guidelines for health-related research (see [EQUATOR Network](http://www.equator-network.org/%20)), life science research (see the [BioSharing Information Resource](https://biosharing.org/)), or the [ARRIVE guidelines](http://www.plosbiology.org/article/info:doi/10.1371/journal.pbio.1000412) for reporting work involving animal research. Where applicable, authors should refer to any relevant reporting standards documents in this form.

If you have any questions, please consult our Journal Policies and/or contact us: [editorial@elifesciences.org](mailto:editorial@elifesciences.org).

**Sample-size estimation**

- You should state whether an appropriate sample size was computed when the study was being designed
- You should state the statistical method of sample size computation and any required assumptions
- If no explicit power analysis was used, you should describe how you decided what sample (replicate) size (number) to use

Please outline where this information can be found within the submission (e.g., sections or figure legends), or explain why this information doesn’t apply to your submission:

No sample size calculation was performed in advance of collecting data, though we retrospectively applied methods to evaluate the statistical significance of our results in retrospect. The extent of data collection was based upon the prior selection of five wards in the hospital with which a particularly high number of infections were linked. The selection of a subset of all of the wards in the hospital was made for pragmatic reasons; our dataset records the locations of health care workers who were infected; these data were collected in the most part retrospectively via the examination of shift data, and by means of speaking to workers individually; a resource-intensive process.

**Replicates**

- You should report how often each experiment was performed
- You should include a definition of biological versus technical replication
- The data obtained should be provided and sufficient information should be provided to indicate the number of independent biological and/or technical replicates
- If you encountered any outliers, you should describe how these were handled
- Criteria for exclusion/inclusion of data should be clearly stated
- High-throughput sequence data should be uploaded before submission, with a private link for reviewers provided (these are available from both GEO and ArrayExpress)

Please outline where this information can be found within the submission (e.g., sections or figure legends), or explain why this information doesn’t apply to your submission:

We report the analysis of a dataset that was not collected via experimental means, so that the concept of replication is not appropriate within the bounds of our study. All analyses were applied to all data collected, with the exception of cases where a subset of the data (e.g. green wards) was of interest. Where for computational reasons a calculation was performed upon a subset of the data, a measure for convergence was used to check the results obtained; this convergence calculation is described in full in our manuscript.

**Statistical reporting**

- Statistical analysis methods should be described and justified
- Raw data should be presented in figures whenever informative to do so (typically when N per group is less than 10)
- For each experiment, you should identify the statistical tests used, exact values of N, definitions of center, methods of multiple test correction, and dispersion and precision measures (e.g., mean, median, SD, SEM, confidence intervals; and, for the major substantive results, a measure of effect size (e.g., Pearson's r, Cohen's d)
- Report exact p-values wherever possible alongside the summary statistics and 95% confidence intervals. These should be reported for all key questions and not only when the p-value is less than 0.05.

Please outline where this information can be found within the submission (e.g., sections or figure legends), or explain why this information doesn’t apply to your submission:

We have reported a p-value and how we obtained it in one case during the paper, giving full information on the calculation in the Methods section. Other than this we made use of the Bayesian information criterion; we have given the appropriate citation for this method rather than describing it in full.

(For large datasets, or papers with a very large number of statistical tests, you may upload a single table file with tests, Ns, etc., with reference to sections in the manuscript.)

**Group allocation**

- Indicate how samples were allocated into experimental groups (in the case of clinical studies, please specify allocation to treatment method); if randomization was used, please also state if restricted randomization was applied
- Indicate if masking was used during group allocation, data collection and/or data analysis

Please outline where this information can be found within the submission (e.g., sections or figure legends), or explain why this information doesn’t apply to your submission:

This is not a clinical study in the sense that we did not compare the effects of different treatments, making only a retrospective evaluation of events. Individuals are grouped in the study only according to the physical ward upon which they were located.

**Additional data files (“source data”)**

- We encourage you to upload relevant additional data files, such as numerical data that are represented as a graph in a figure, or as a summary table
- Where provided, these should be in the most useful format, and they can be uploaded as “Source data” files linked to a main figure or table
- Include model definition files including the full list of parameters used
- Include code used for data analysis (e.g., R, MatLab)
- Avoid stating that data files are “available upon request”

Please indicate the figures or tables for which source data files have been provided:

We have provided source data for figures 1, 2S2, 4, 4S1, 4S3, 4S5, 5S1, 5S2, and 5S5. These describe the raw data in each figure and in some cases the data underlying what is shown in the figure e.g. other likelihoods where a maximum likelihood outcome is shown. Our inference code has been made available via Github.
